# Bench-Top Fabrication of an All-PDMS Microfluidic Electrochemical Cell Sensor Integrating Micro/Nanostructured Electrodes

**DOI:** 10.3390/s17040732

**Published:** 2017-03-31

**Authors:** Sokunthearath Saem, Yujie Zhu, Helen Luu, Jose Moran-Mirabal

**Affiliations:** Department of Chemistry and Chemical Biology, McMaster University, 1280 Main Street West, Hamilton, ON L8S 4M1, Canada; saems@mcmaster.ca (S.S.); zhuy8@mcmaster.ca (Y.Z.); luuh2@mcmaster.ca (H.L.)

**Keywords:** fibroblast, shape memory polymer, flexible biosensor, cyclic voltammetry, cell sensor, xurography, stencil lift-off, on-chip electrochemical sensor

## Abstract

In recent years, efforts in the development of lab-on-a-chip (LoC) devices for point-of-care (PoC) applications have increased to bring affordable, portable, and sensitive diagnostics to the patients’ bedside. To reach this goal, research has shifted from using traditional microfabrication methods to more versatile, rapid, and low-cost options. This work focuses on the benchtop fabrication of a highly sensitive, fully transparent, and flexible poly (dimethylsiloxane) (PDMS) microfluidic (μF) electrochemical cell sensor. The μF device encapsulates 3D structured gold and platinum electrodes, fabricated using a shape-memory polymer shrinking method, which are used to set up an on-chip electrochemical cell. The PDMS to PDMS-structured electrode bonding protocol to fabricate the μF chip was optimized and found to have sufficient bond strength to withstand up to 100 mL/min flow rates. The sensing capabilities of the on-chip electrochemical cell were demonstrated by using cyclic voltammetry to monitor the adhesion of murine 3T3 fibroblasts in the presence of a redox reporter. The charge transfer across the working electrode was reduced upon cell adhesion, which was used as the detection mechanism, and allowed the detection of as few as 24 cells. The effective utilization of simple and low cost bench-top fabrication methods could accelerate the prototyping and development of LoC technologies and bring PoC diagnostics and personalized medicine to the patients’ bedside.

## 1. Introduction

Sustainable global healthcare is a long sought-after idea [1]. Innovation in modern health care diagnostic techniques continues to improve patient outcomes, but the cost of bringing the technology to patients worldwide increases concomitantly. Ever since the inception of the portable glucose meter, scientists and engineers have been interested in lab-on-a-chip (LoC) devices for point-of-care (PoC) detection as low-cost and portable solutions to health screening, diagnostics, and personalized medicine [2,3]. To fabricate such devices on a mobile platform, LoC technology requires an all-in-one solution comprised of sensing and conducting elements within a microfluidic channel of sub-millimeter dimension [4].

Microfabricated on-chip microfluidic electrochemical biosensors have the advantage of being label-free, and offer high sensitivity and quantitative detection of analytes over a much broader concentration range than their fluorescence or colorimetric counterparts [4,5,6,7,8,9]. Most traditional microfabrication techniques are inherited from the semiconductor industry, where lithography [10,11,12], thin film deposition [12,13], and etching are routinely employed [12,14]. These techniques are very effective at producing high-resolution patterns at the micro- to nanoscale on a wide variety of materials. Yet, traditional microfabrication approaches can require expensive equipment, access to cleanroom facilities, multistep processes, and often limit themselves to rigid and non-transparent substrates. Some alternatives to making on-chip μF biosensors take advantage of soft silicone elastomers (e.g., poly (dimethylsiloxane)—PDMS) bonded to glass, silicon wafers, or polystyrene sheets through bonding techniques such as plasma oxidation and surface chemical grafting [13,15,16,17,18]. These methods offer a cost-effective bench-top alternative for the fabrication of μF devices without the use of expensive cleanroom facilities. Furthermore, μF devices for applications such as conformal pressure sensors, and flexible and transparent electronics, need to be implemented on soft and flexible materials, which sometimes cannot be processed through traditional methods. To overcome the limitations of cost, time, material properties, and processing, non-traditional techniques are being explored, including xurography [13,15,18], adhesive stencil lift off [15,18,19,20], shape-memory polymers (SMP) [13,15,20,21,22], ink jet printing [23,24], paper-based microfluidic devices [5,7,25,26,27], and 3D printing [28,29]. Despite the reduced resolution when compared to traditional microfabrication, these modern techniques offer lower costs and shorter turnaround time, two important factors for rapid prototype development and commercial scalability.

Recently, SMPs have gained significant traction in the microfabrication community for their ability to produce 3D micro/nanostructured surfaces on a variety of thin films (e.g., Au, Pt, CNT, SiO_2_, and TiO_2_) [13,20,30,31,32,33,34,35]. The structured surfaces are produced through a compressive stress applied by the SMP during shrinking once it is heated over its glass transition temperature [21,36]. This technique has been shown to successfully structure gold films for use as electrodes for electrochemical sensing [13,15,20,37] and as substrates for surface-enhanced Raman scattering [13,38,39,40]. The micro/nanostructures described in the literature showed increased electroactive surface area for working electrodes, ideal for sensing within μF devices. This simple bench-top fabrication approach presents an attractive route for the integration of highly sensitive electrochemical techniques into flexible LoC microfluidic devices.

This work presents a benchtop, low-cost, and rapid method for fabricating a highly sensitive, all-PDMS, μF electrochemical sensor, and demonstrates its sensing capabilities through the detection of the adhesion of murine 3T3 fibroblast cells—the most abundant cells in connective tissue and critical components in wound healing—onto the surface of the electrode. Commercial PDMS elastomer and pre-stressed polystyrene were used as the bulk substrates for the μF device and to fabricate the 3D micro/nanostructured electrodes, respectively. A salt-bridge-free three-electrode electrochemical μF device was fabricated to encapsulate the structured gold working, auxiliary, and platinum reference electrodes for an all-PDMS μF sensor. Cyclic voltammetry was employed to study the flow rate effects on charge transfer efficiency of the structured electrodes. Finally, fibroblasts were incubated and adhered onto the structured electrode and sensed using a redox reporter. This work demonstrates proof-of-concept for a novel rapid prototyping method to fabricate a transparent flexible sensor possessing high sensitivity and reproducibility. We anticipate that further surface functionalization of the structured electrodes will lead to inexpensive devices for the label-free detection of specific cell types, DNA-aptamer binding, and electrochemical or fluorescence sensing for applications in personalized precision medicine and PoC diagnostics.

## 2. Materials and Methods

### 2.1. Electrode Fabrication

**Figure 1:**
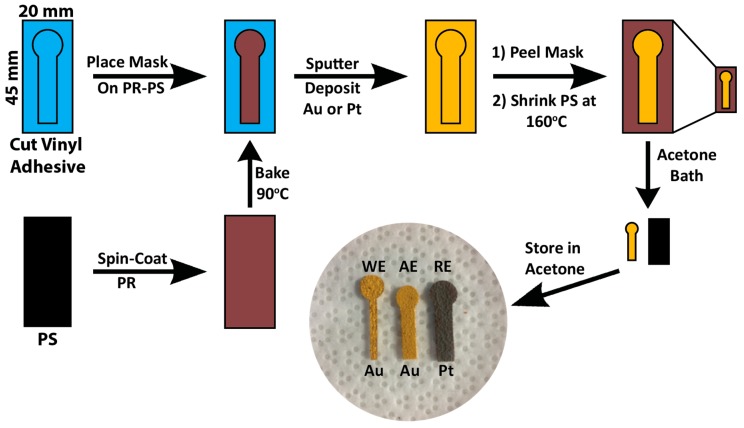
Schematic of the bench-top fabrication method for the patterning, structuring and lift-off of the working, auxiliary, and reference electrodes (WE, AE, RE). Electrodes were patterned by cutting vinyl adhesive stencils (blue) to the desired shape and placing them on photoresist (PR, maroon) coated pre-stressed polystyrene (PS, black). Gold (100 nm) and platinum (150 nm) were sputtered onto the masked substrates followed by removal of the vinyl stencils. The sputtered flat electrodes were then subjected to heat at 160 °C to shrink the PS substrate down to 16% of its original area. The shrinking process resulted in Au and Pt micro/nanostructured electrodes, which were then lifted off by dissolving the PR in an acetone bath.

The structured electrodes used within the microfluidic device were fabricated using a vinyl stencil lift-off and pre-stressed polystyrene (PS) shrinking method previously described (Figure 1) [20]. Briefly, the PS sheets (Shrink Film, Graphix, Maple Heights, OH, USA) were cut to the desired shape using a blade cutter (ROBOPro CE5000-40-CRP, Graphtec America Inc., Irvine, CA, USA) followed by a 3-step wash with isopropyl alcohol, ethanol, and 18.2 MΩ water under constant agitation at 60 rpm, and dried under a nitrogen (N_2_) stream. The clean PS sheets were spin-coated with a positive-tone photoresist (PR, Shipley 1818, Marlborough, MA, USA) layer with a 1.8 μm nominal thickness. The PR-coated PS sheets were then baked on a hot plate at 90 °C for 3 min to remove residual solvent. The self-adhesive vinyl (FDC-4300, FDC graphics films, South Bend, IN, USA) shadow masks were also patterned using the blade cutter to the specific electrode dimensions for the working (WE) auxiliary (AE) and reference electrodes (RE), which were set to 10.8 mm × 1.6 mm × 4.8 mm, 10.8 mm × 3.0 mm × 4.8 mm, and 10.8 mm × 3.0 mm × 4.8 mm (length × width × pad diameter), respectively (Figure S1). The adhesive vinyl masks were then transferred onto the clean PS and served as stencils during the metal deposition process. Gold (99.999% purity, LTS Chemical Inc., Chestnut Ridge, NY, USA) and platinum were deposited using a Torr Compact Research Coater CRC-600 manual planar magnetron sputtering system (Torr International, New Windsor, NY, USA) at deposition rates of ~1 Å/s (100 nm) and ~0.1 Å/s (150 nm), respectively. Following this step, the vinyl stencils were lifted off and the PS sheets were placed in an oven heated at 160 °C, which shrunk the PS substrates to ~16% of their original size by area [15,20]. The structured metal films were lifted off from the PS by dissolving the PR in an acetone bath under constant agitation at 80 rpm for 30 min. Once the electrodes were lifted off from the PS, they were stored in acetone until further use.

### 2.2. All PDMS μF Device Fabrication

**Figure 2:**
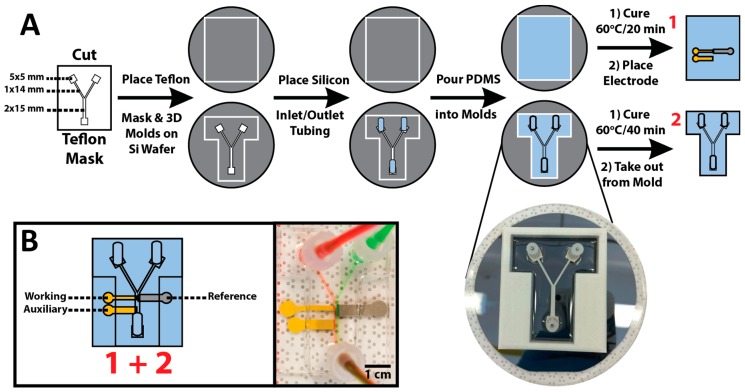
Bench-top microfabrication process for making the all-PDMS on-chip μF electrochemical biosensors. (**A**) A Teflon adhesive μF channel mold was cut and placed inside a 3D printed device mold on top of a Si-wafer to ensure surface flatness. Silicon tubing was placed at the inlet/outlet positions, and PDMS was poured into the mold until full. The bottom layer (1) was partially cured at 60 °C for 20 min followed by placement of the three electrodes. The top layer (2) was cured at 60 °C for 40 min and removed from its mold. (**B**) PDMS Layers (1) and (2) were plasma treated for 30 s, bonded, and allowed to fully cure at 60 °C for 1 h.

The μF channel mold was patterned using the blade cutter into a Bytac^®^ PTFE surface protection laminate (Sigma-Aldrich, St. Louis, MO, USA) adhesive film with 125 μm nominal thickness. The patterned mold dimensions are shown in Figure 2a. 3D printed corrals defining the size of the PDMS μF layers (white, Figure 2a) were fabricated out of acrylonitrile butadiene styrene polymer using a Replicator 2X Experimental 3D Printer (MakerBot Industries, Brooklyn, NY, USA). The PTFE adhesive channel mold and 3D printed corral were placed on top of a 3-inch Si wafer and silicone tubing (Masterflex, Gelsenkirchen, Germany) was placed on top of the inlet and outlet reservoirs of the PTFE mold. A Sylgard-184 (Dow Corning, Midland, MI, USA) elastomer and hardener were mixed in a 10:1 ratio, degassed for 20 min, and poured into the 3D printed corrals. The top PDMS layer (2, Figure 2a) was placed first in an oven at 60 °C for 20 min followed by the bottom layer (1, Figure 2a) for the remaining 20 min before both halves were taken out of the oven and allowed to cool to room temperature. The structured Au [working (WE) and auxiliary (AE)] and Pt [reference (RE)] electrodes were placed on top of PDMS Layer 1. PDMS Layer 2 was removed from the 3D mold, and both layers were treated with air plasma for 30 s (30 sccm air inlet flow and 600 mTorr pressure) at a high-power setting (30 W) in a PDC expanded plasma cleaner (Harrick, Ithaca, NY, USA). The two layers were then bonded and left to fully cure at 60 °C for 1 h (Figure 2b).

### 2.3. Dead-End Pressure Test of PDMS Bond Strength

All dead-end pressure tests were performed in triplicate for each bonding condition. A dead-end chamber with dimensions of 7 mm × 7 mm × 0.125 mm was made using patterned square PTFE molds as described above. The dead-end devices were attached to a N_2_ (g) source with an inline pressure gauge and placed in a beaker filled with water. The N_2_ (g) flow was increased until the water began to bubble, indicating that the device had burst through delamination or mechanical failure (Video S1 and Figure S2).

### 2.4. Murine 3T3 Fibroblast Cell Culture

Murine 3T3 fibroblast cells were prepared through standard cell culturing procedures. Briefly, Dulbecco’s modified eagle’s medium (DMEM, supplemented with 10% FBS, 1% L-glutamine, 1% penicillin/streptomycin (Invitrogen, Life Technologies, Burlington, ON, Canada)) was heated to 37 °C and 30 mL were pipetted into a 50 mL conical tube kept at 37 °C. A Frozen cryovial of murine 3T3 fibroblasts (ATCC, Manassas, VA, USA) was rapidly thawed by swirling the contents in a 37 °C water bath. To remove DMSO (Caledon Laboratory Chemicals, Georgetown, ON, Canada) from the cryovial contents, the contents were poured into the media kept at 37 °C and centrifuged at 500 g for 5 min. The supernatant was discarded, and the cell pellet was re-suspended in 15 mL of supplemented DMEM and incubated overnight in a T25 tissue culture flask (Sigma-Aldrich, Oakville, ON, Canada). Once the cells had grown to the desired confluency, they were detached from the culture flask by adding a trypsin solution (TrypLE, Thermo Fisher, Waltham, MA, USA) at 37 °C for 3–5 min. To neutralize the trypsin, an equal amount of DMEM was added (supplemented with 10% Fetal Bovine Serum and 1% Penicillin—both from Thermo Fisher, Waltham, MA, USA). The cell solution was then pipetted into a centrifuge tube and centrifuged at 500 g for 5 min followed by extraction of the supernatant. The cells were re-suspended with the desired amount of DMEM and counted using a hemocytometer cell counter and a light microscope. Finally, the cell solution was diluted to 2 × 10^6^ cells/mL using DMEM and stored at 37 °C and 5% CO_2_(g) until use in the μF cell incubation step.

### 2.5. CFSE Fibroblast Cell Staining

A solution containing 50 μM CFSE (5(6)-carboxyfluorescein diacetate N-succinimidyl ester, Sigma-Aldrich, Oakville, ON, Canada) was prepared from a 1 mM stock and 0.1% FBS in 1× PBS (phosphate buffer saline, 100 mM, pH = 7.4). Following this, 2 × 10^6^ fibroblast cells were added to 200 μL of the CFSE solution, gently mixed and incubated at 37 °C in the dark for 1 h. After the incubation period, 200 μL of 100% FBS was added to the mixture and incubated at 37 °C for 10 min in the dark. The mixture was then centrifuged at 500 g for 5 min with the resulting pellet being washed 3 times with 10% FBS in 1× PBS solution. Finally, the pellet was re-suspended to the desired concentration in DMEM.

### 2.6. Cell Viability Assay

The viability assay for Au-PDMS substrates was carried out with PrestoBlue (Invitrogen, San Diego, CA, USA) in a 48-well plate. To start, 10^4^ fibroblasts in 1 mL of DMEM were plated in each of the 12 wells and incubated at 37 °C/5% CO_2_ for 24 h to ensure optimal cell adhesion. Among these, 4 wells contained Au-PDMS substrates, 4 were used to test 1 h 1× PBS incubation, and the last 4 were used for the DMEM control. For the viability assay, the media in all 12 wells was substituted with 180 μL of 1× PBS and 20 μL of 10x PrestoBlue solution. After 30 min of cell incubation at 37 °C and 5% CO_2_, the change in the fluorescence of the samples was measured using a Cytation multi-well plate reader (Biotek Instruments Inc., Montreal, QC, Canada) with the excitation/emission wavelengths set at 535/615 nm.

### 2.7. Murine 3T3 Fibroblast Cell Sensing

The Au electrodes (WE and AE) in the PDMS μF device were preconditioned and baselined through cyclic voltammetry (CV). A 100 mM H_2_SO_4_ working solution and a 1× PBS reference solution were pumped through the μF device in separate laminar streams at 0.1 mL/min using a PHD ULTRA™ Syringe Pump (Harvard Apparatus, Holliston, MA, USA). The devices were preconditioned through 30 CV scans for the working electrode performed from 0.0 to 1.4 V at a scan rate of 0.10 V/s using a CHI600E electrochemical workstation (CH Instruments, Austin, TX, USA). Following the preconditioning step, the entire μF channel was washed with 1× PBS for 5 min at 0.10 mL/min. Prior to cell incubation, a baseline CV scan was performed on each μF device, yielding the total current for the clean WE. A sensing solution of 5 mM K_4_[Fe(CN)_6_] in DMEM was continuously pumped through the device at 0.1 mL/min, and a 10 segment CV scan was performed from −0.4 to 0.2 V (with respect to the RE) at a scan rate of 0.10 V/s. Once again, the device was washed by flowing 1× PBS for 5 min at 0.1 mL/min. Finally, a fibroblast solution containing 2 × 10^6^ cells/mL in DMEM was pumped through the μF stream flowing over the WE at 0.1 mL/min for 1 min and then stopped to allow for fibroblast attachment onto the WE for 1 h at 37 °C and 5% CO_2_(g). After cell adhesion, the μF device was flushed with the sensing solution, and CV scans were performed as described above. Experiments were performed on three replicate devices to assess reproducibility and the statistical significance of the results.

### 2.8. Fluorescent Microscopy Image Acquisition

The murine 3T3 fibroblast cells inside the μF channel were imaged using a Nikon Eclipse LV100N POL epifluorescence microscope (Nikon Instruments, Mississauga, ON, Canada) equipped with excitation and emission filters for FITC dye, and a Nikon MRP50102 10×/0.25 NA Pol objective. Images were acquired with a Retiga 2000R cooled CCD camera (QImaging, Surrey, BC, Canada) and recorded with NIS-Elements AR software (Nikon Instruments, Mississauga, ON, Canada). The images were taken at 200 ms exposure time, 2 × 2 binning, and a hardware gain of 10.

## 3. Results and Discussion

### 3.1. Optimization of PDMS Device Bond Strength for μF Device Fabrication

**Figure 3:**
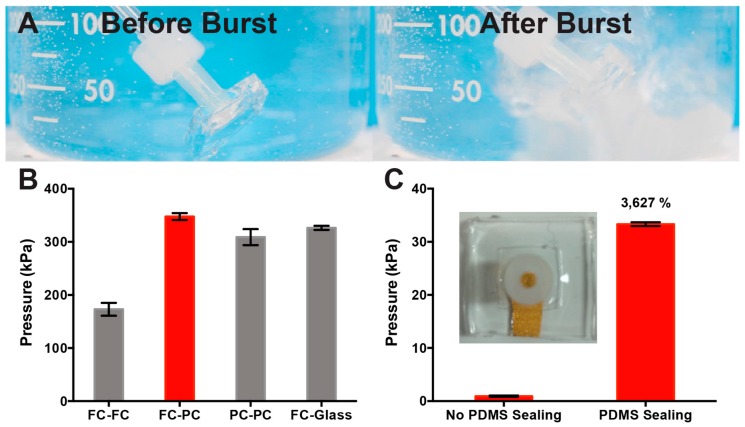
PDMS bonding strength characterization All PDMS curing was done in a 60 °C oven with the following curing times as follows: fully cured (FC)—40 min; partially cured (PC)—20 min. (**A**) Image of the burst pressure test with the intact dead-end μF device (left) and the broken device (right). (**B**) Quantification of the burst pressure for different bonding combinations of FC–FC PDMS layers, PC–FC PDMS layers, PC–PC PDMS layers, and FC PDMS layer-glass. (**C**) Quantification of the burst pressure for a dead-end μF device with a 5 mm Au electrode in place. Graph compares devices without PDMS sealing around the device vs. with PDMS sealing after bonding. The inset shows a sample image of the dead-end burst pressure device with the Au electrode in place as would be seen in the μF device. All error bars represent the standard error of the mean, *n* = 3.

A viable flexible μF sensing platform must match or surpass current μF solutions in reproducibility, sensitivity, and reliability. The challenge in having a flexible all-PDMS μF sensor is that its intrinsic mechanical flexibility can introduce delamination at the bonding interface between the two PDMS layers of the μF device as it is being handled. In particular, the PDMS device should be strong enough to withstand the pressures generated from pressurized flow through the microfluidic channel. Therefore, the characterization and optimization of the bond strength between the two layers of a silicone-based μF device is crucial for the production of a reliable and flexible μF sensing platform. The bond strength of various bonding conditions was quantified using a dead-end chamber pressure test. To perform such a test, triplicate devices with dead-end chamber dimensions of 7 mm × 7 mm × 0.125 mm were made as described in the Materials and Methods section, and the bond strength was measured by pressurizing gas into the dead-end device until either the device delaminated or the PDMS layer/inlet tubing burst. The burst pressure was actively monitored using an inline pressure gauge. Performing the burst pressure test with the devices submerged in water provided an immediate indication of the device failure, as the escaping pressurized gas produced vigorous bubbling in the solution (Figure 3a, Video S1). Figure 3b shows a comparison of the burst pressure for different PDMS–PDMS bonding conditions benchmarked against the standard PDMS–glass interface, and a comparison of a device integrating a structured electrode 5 mm in width with and without PDMS sealing at the electrode–PDMS interface.

The bonding conditions depicted in Figure 3b for PDMS devices without a structured electrode differ only in the combination of PDMS curing time (partially cured–PC vs. fully cured–FC), with every treatment subjected to 30 s air plasma treatment prior to device bonding. PC PDMS was made by subjecting the PDMS to 60 °C for 20 min and fully cured FC PDMS was subjected to 60 °C for 40 min prior to bonding followed by a final curing step at 60 °C for 1 h. The devices were allowed to form stable bonds at room temperature for 24 h after the initial bonding. FC–FC PDMS bonding exhibited an average burst pressure of 170±40 kPa, which was ~50% lower than the FC–glass benchmark of 330±10 kPa. The FC–PC and PC–PC combination produced statistically equivalent dead-end burst pressures to the benchmark, failing at 350±20 and 310±50 kPa, respectively. The increased bond strength observed in the FC–PC and PC–PC compared to the FC–FC devices can be attributed to having both hydrosilylation and dehydration reactions occurring at the interface. The hydrosilylation reaction is facilitated by a Pt catalyst found in the Sylgard-184 cross-linker and occurs when free silicon hydride (SiR_3_-H) groups found in the Sylgard-184 elastomer base crosslinks with the vinyl-terminated polysiloxanes found in the Sylgard-184 cross-linker. Hydrosilylation can only occur with the partially cured PDMS mixture, where silicone chains are relatively mobile, while the condensation reaction occurs between two adjacent silanol (SiR_3_–OH) groups, which will react to form Si–O–Si bonds and eliminate H_2_O as a by-product. By exposing the two PDMS surfaces to plasma oxidation, we can generate surface SiR_3_–OH, which will contribute to strengthening the PDMS–PDMS bonding interface over time. In addition, using the PC PDMS, the surface roughness effects on bonding are minimized, since the PC surface is still moldable and can conform to the complementary bonding layer. In view of the bonding strength test results for all-PDMS layers, we chose to use the FC–PC combination for the fabrication of devices incorporating structured electrodes, since it has a comparable failure pressure to the FC PDMS–glass benchmark, is relatively easy to handle, and has higher reproducibility than the FC–FC combination. Optimization of the bonding with layers containing structured electrodes was done using the same dead-end layout, but with the integration of a structured electrode that covered ~70% of one of the side walls (inset, Figure 3c). This setup was chosen to test the disruption in bonding strength with an extreme case of electrode-to-sidewall ratio. As anticipated, once a structured electrode was in place, the bonding strength was much weaker (0.9±0.1 kPa, Figure 3c) than that observed with the all-PDMS FC–PC combination. To overcome such weak bonding, we resorted to sealing the device edges with freshly mixed PDMS after the initial bonding, with the intent to reinforce the device bond at the site of the electrode. By sealing the device with the PDMS mix and then curing, the device burst pressure increased >36-fold (33.0±0.4 kPa, Figure 3c) when compared to the devices without sealing. This technique was then used on the μF device, and the burst flow rate was determined. With the PDMS sealing, the burst flow rate was determined to be >100 mL/min (maximum capability of the syringe pump). Thus, the bond strength with the optimized fabrication procedure is more than suitable for experimentation, where flow rates >4 mL/min have been shown to detach murine 3T3 fibroblast cells incubated within μF devices of similar dimensions without the use of fibronectin as an adhesion promoter [41].

Using FC–PC bonding and sealing procedure, all the subsequent PDMS μF devices were made in accordance to the process depicted in Figure 2a. The challenges in fabricating such devices with high reproducibility were in the careful placement of the three electrodes in the appropriate configuration, and the successful bonding over the structured electrode surfaces. It was found that following the 20 min curing step for the bottom half of the μF device, the three electrodes had to be immediately transferred from the acetone storage solution to the PDMS to take full advantage of the tackiness of the PC–PDMS for maximal electrode-to-PDMS adhesion. Once the electrodes had made contact with the PC–PDMS, the adhesion became too strong to remove the electrodes without destroying them. Since the transfer must be done successfully in one attempt, placing the electrodes one by one would require very high dexterity and precision to fabricate μF devices with acceptable reproducibility. To overcome this challenge, a Teflon filter membrane was used as a base support for the configuration and transfer of the electrodes in a single step. To ensure that the electrodes adhered to the Teflon membrane and that the membrane did not stick to the PC–PDMS, the membrane was pre-wet with a small amount of acetone (this also afforded transparency through the Teflon membrane for optimal electrode configuration). The electrodes were then removed from the storage solution using flat tweezers and placed on the membrane. At this stage, the electrodes could be easily positioned into the appropriate configuration without damaging them. Once in position, the electrodes were picked up by the Teflon membrane and placed on top of the PC–PDMS. Upon electrode to PC–PDMS contact, the membrane was then quickly removed. The final steps in the assembly and bonding of the μF devices were performed as described in the Materials and Methods section and depicted in Figure 2a.

### 3.2. Impact of Flow Rate on Electrochemical Sensing

Once the fabrication protocol for the μF devices was optimized, we turned our attention to the electrochemical sensing stability within the devices. Throughout this study, we utilized cyclic voltammetry (CV), a simple electrochemical technique that is commonly used to quantify redox processes and can be leveraged to implement cell-sensing strategies. CV is a diffusion-limited technique when the sensing of the redox process is performed in an unstirred solution, such that the analytes are not disturbed as they are undergoing reduction-oxidation cycles. Performing CV measurements in a flow cell, such as a μF channel, with variable flow rates could mimic a stirred cell condition resulting in limitations to charge transfer efficiencies across the working electrode, thus reducing the overall device sensitivity.

To perform on-chip electrochemical sensing and assess the impact of the flow rate on electrochemical sensing, a salt-bridge-free three electrode system was fabricated to incorporate Au WE and AE, and Pt RE in a Y-shaped bench-top fabricated PDMS μF device (cf. Figure 2b). The salt-bridge-free μF design exploits the laminar flow within the microfluidic channel to isolate the RE from the working sample solution, while still allowing the diffusion of the supporting electrolyte across the laminar flow interface. The separation between the working solution and the reference solution is critical in the stability of the potential output during sensing through CV. Given than the structured Pt film is used as a hydrogen pseudo-reference electrode, its half-cell potential is dependent on the concentration of hydronium ions (i.e., pH). To prevent fluctuations in pH, a buffer solution (1× PBS, pH = 7.4) was pumped through the reference inlet to be in constant contact with the RE, thus maintaining a constant reference electrode potential.

**Figure 4:**
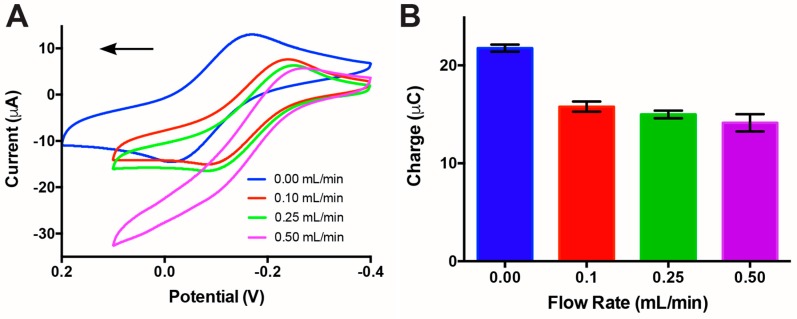
Assessment of the impact of flow rate on charge transfer efficiency. The cyclic voltammetry experiments were performed using a 5 mM K_4_[Fe(CN)_6_] working solution at a 0.10 V/s scan rate. (**A**) Cyclic voltammograms showing changes in reduction and oxidation peaks and down-right shifting of the voltammograms as flow rate increased from 0 to 0.5 mL/min with a positive initial scan direction as indicated by the arrow. (**B**) Quantification of the total charge transfer in the cathodic peak for the different flow rates. Between flow rates of 0.1 and 0.5 mL/min, there is no statistical difference in charge transfer efficiency.

To assess the impact of the working flow rates used for sensing on charge transfer, we performed a series of CV measurements in the μF devices at flow rates from 0–0.5 mL/min. The CV for each flow rate was obtained in a 5 mM K_4_[Fe(CN)_6_] in 1× PBS working solution and a 1× PBS reference solution utilizing the laminar flow inside the μF channel to separate the working and reference solutions (demonstrated in Video S2 using coloured solutions). Figure 4a shows an overlay of the cyclic voltammograms for the different flow rate conditions, with cathodic peaks shifting from −0.22 to −0.18 V with respect to stopped flow (blue curve, 0 mL/min). To perform the stopped flow sensing, the solution was pumped into the μF device, then the flow was stopped, and CV scans were then performed. The voltammogram shows a change in CV curve shape from a classic “duck” shape to a sigmoidal shape from low to high flow rates. The change in shape indicates that at higher flow rates we begin to reach a limit at which the convection introduced by the laminar flow is much greater than the redox kinetics of K_4_[Fe(CN)_6_]. The result is non-uniform anodic peak current to cathodic peak current and ultimately a change from a completely reversible process to a quasi-reversible process. Despite the signal shifts, the integration of the reduction peaks (Figure 4b) provides a quantification of the charge transfer, showing that there is an initial reduction in total charge to ~73% compared to the stopped flow. Between the flow rates of 0.1 and 0.5 mL/min, there is no statistical difference in charge transfer efficiencies. After confirming that the working flow rates (0.1–0.5 mL/min) did not negatively impact the electrochemical sensing capabilities of the PDMS μF device, we chose the lowest working flow rate tested of 0.10 mL/min for the murine 3T3 fibroblast cell sensing. This minimized the risk of cell detachment, while maintaining a continuous flow within the μF device. Minimizing cell detachment during the sensing period is essential during the acquisition of the voltammograms, since any such event would cause a significant increase in charge transfer efficiency at the working electrode.

### 3.3. On-Chip Cell Detection

**Figure 5:**
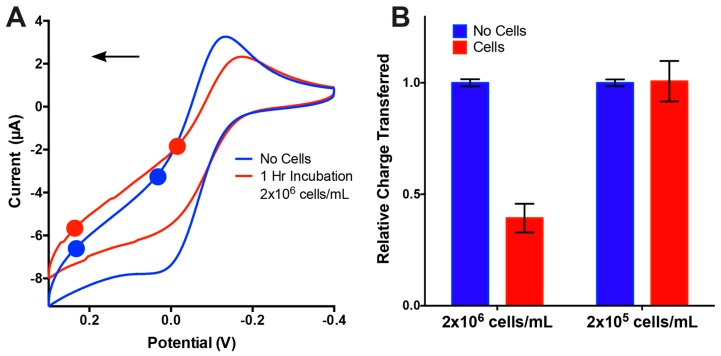
Fibroblast sensing in 5 mM K_4_[Fe(CN)_6_] solution at a 0.1 mL/min flow rate. (**A**) Cyclic voltammogram of the detection of murine 3T3 fibroblast cells vs. control electrode. (**B**) Relative charge transferred by the working electrode during the detection of murine 3T3 fibroblast cell vs. the control electrode. All error bars represent the standard error of the mean, *n* = 3.

To evaluate the impact of DMEM on electrochemical sensing over the incubation time, CV was performed immediately after being exposed to DMEM, and in DMEM after incubation for 1 h. We observed no statistical difference between the voltammograms obtained in DMEM immediately after exposure and those obtained after 1 h of incubation (Figure S4). Prior to on-chip cell detection, the murine 3T3 fibroblast cells were introduced into the μF channel by pumping through a solution containing 2 × 10^6^ cells/mL in DMEM at 0.1 mL/min for 1 min, stopping the flow, and allowing the cells to attach to the WE by incubating them in the device for 1 h at 37 °C. The total volume of the device was ~2 μL, which translates into ~4000 fibroblast cells within the device at any given time. The experiment was performed on triplicate devices on the same day within a 4 h timeframe to minimize variability in cell adhesion and sensing due to ageing of the cells. After cell incubation, the μF was washed by pumping it through DMEM at 0.1 mL/min for 2 min to remove any weakly bound or unbound cells from the electrode surface. Using the minimal flow rate of 0.1 mL/min, the adhered murine 3T3 fibroblast cells were sensed in a DMEM working solution supplemented with 5 mM K_4_[Fe(CN)_6_]. During cell sensing, the redox-reporter was monitored through CV, where Figure 5a presents a typical voltammogram showing the cathodic peaks for a device before (blue) and after 3T3 cell incubation (red). The cells inside the μF device were monitored throughout the adhesion steps with fluorescence microscopy and a live CFSE cell stain, which can be seen in Figure S3. The figure shows images comparing the number of cells located on the Au–WE before incubation, after incubation (washed), and after sensing vs. control plain Au–WE. After sensing, the average number of cells counted on the WE (0.8 mm×1.6 mm geometric area) was ~24 cells.

To quantify the adhesion of cells to the electrode, the cathodic peaks of the CVs were integrated and the total charge transferred calculated. For the cathodic peak integration, the baseline correction was done through a linear regression using two points seen on each cyclic voltammogram (Figure 5a). To avoid any integration bias, the first point was placed at the initial leveling during the reduction sweep and the second point was placed at the base of the reduction peak. Upon integrating the cathodic peaks (Figure 5b), a 61% loss of charge transfer was recorded after incubation for the $2\;\times \;10^{6}$ cell/mL solution. When the cell concentration was reduced by an order of magnitude, the electrochemical signal did not change. We attribute this to the low cell concentration in the seeding solution, where in the absence of adhesion proteins like fibronectin, we would expect one or two cells to adhere to the electrode area. This number of cells might be too low to produce a significant change in the electrochemical signal. The CV results show that the PDMS μF sensors were successful in detecting cell adhesion from murine 3T3 fibroblast cells at a relatively low number of cells (~24) and in a low sample volume. The sensitivity of the all-PDMS μF biosensor can be attributed to the use of structured working electrodes. Micro/nanostructuring of deposited thin metal films electrodes through the use of shape-memory-polymers has previously been shown to increase the electroactive surface area per geometric area by ~600% when compared to their flat counterparts [30]. Thus, the area blocked by any individual adhered cell is 6-fold higher than it would be for a traditional flat electrode. While we have demonstrated the capability of detecting murine 3T3 fibroblast cells, the described proof-of-concept detection approach is not specific. Our current efforts are directed towards the functionalization of the structured electrodes with capturing agents that selectively bind targeted cells. This will lead to a reduction in sensing time, improved sensitivity, and reproducibility.

## 4. Conclusions

This work demonstrates a novel, rapid, and cost-effective method for the integration of 3D structured high surface area electrodes into all-PDMS μF devices. The use of xurography on vinyl, PTFE, and PS and 3D printing offers a simple approach for the rapid and inexpensive prototyping and mold fabrication for μF devices. The optimized PDMS–PDMS bonding and sealing conditions offer bond strengths that allow the operation of the microfluidic devices under high flow rates and offer long-lasting encapsulation of the high surface area structured electrochemical sensors. In addition, the μF devices showed sustained charge transfer that was minimally disturbed by relatively low flow rates, as shown through cyclic voltammetry. The on-chip electrochemical sensing capabilities of bench-top fabricated μF devices were demonstrated by sensing the adhesion of model murine 3T3 fibroblast cells, where the signal decreased ~61% after the adhesion of an average of 24 cells over the working electrode. This highlights the excellent sensitivity of the structured Au electrodes. The bench-top fabrication method presented in this work offers a rapid, reproducible, and highly sensitive prototyping method that shows promise for the future prototyping of flexible devices for portable point-of-care diagnostics and personalized medicine.

## Supplementary Materials

The following materials are available online at http://www.mdpi.com/1424-8220/17/4/732/s1: Figures S1–S6, Video S1 and Video S2.
