# Supplementary material for: Bench-Top Fabrication of an All-PDMS Microfluidic Electrochemical Cell Sensor Integrating Micro/Nanostructured Electrodes
Source: Sensors (Basel). 2017 Mar 31;17(4):732. doi: 10.3390/s17040732 (PMC5421692; doi:10.3390/s17040732)
Supplement: Supplementary file 1 [file sensors-17-00732-s001.zip › sensors-17-00732-suppl.pdf]

sensors

MDPI

Supplementary Materilas

Bench-Top Fabrication of an All-PDMS Microfluidic Electrochemical Cell Sensor Integrating Micro/Nanostructured Electrodes

Sokunthearath Saem, Yujie Zhu, Helen Luu and Jose Moran-Mirabal *

Department of Chemistry and Chemical Biology, McMaster University, 1280 Main Street West, Hamilton, ON L8S 4M1, Canada; saems@mcmaster.ca (S.S.); zhuy8@mcmaster.ca (Y.Z.); luuh2@mcmaster.ca (H.L.)
* Correspondence: mirabj@mcmaster.ca; Tel.: +1-905-525-9140

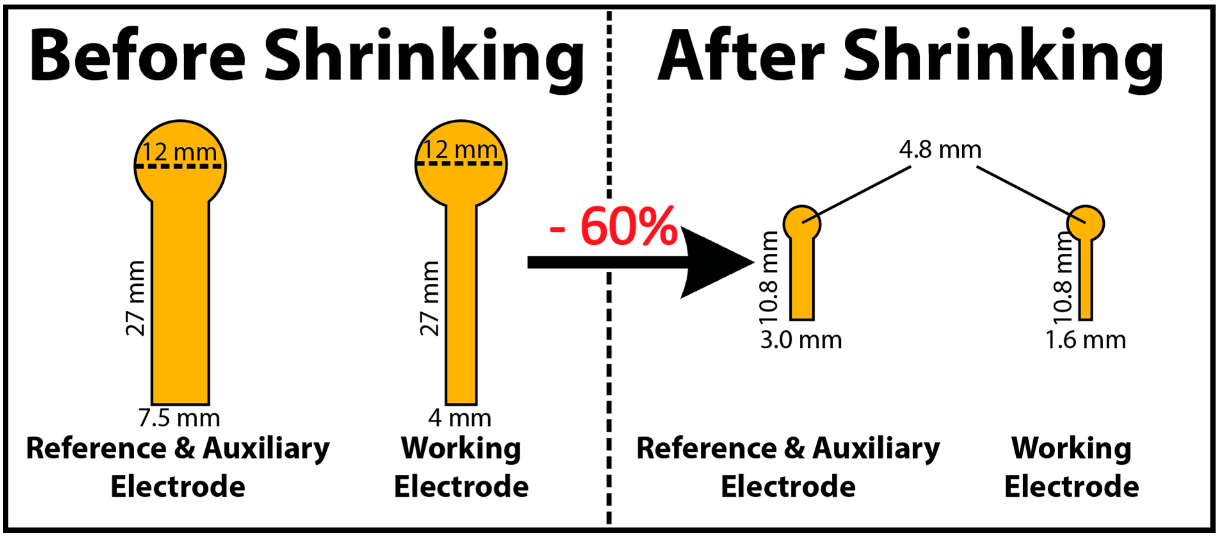

Figure S1. Schematic of the dimension of the electrodes used in the microfluidic device. On the left shows the relative size comparison of the electrodes prior to shrinking to after shrinking (right).

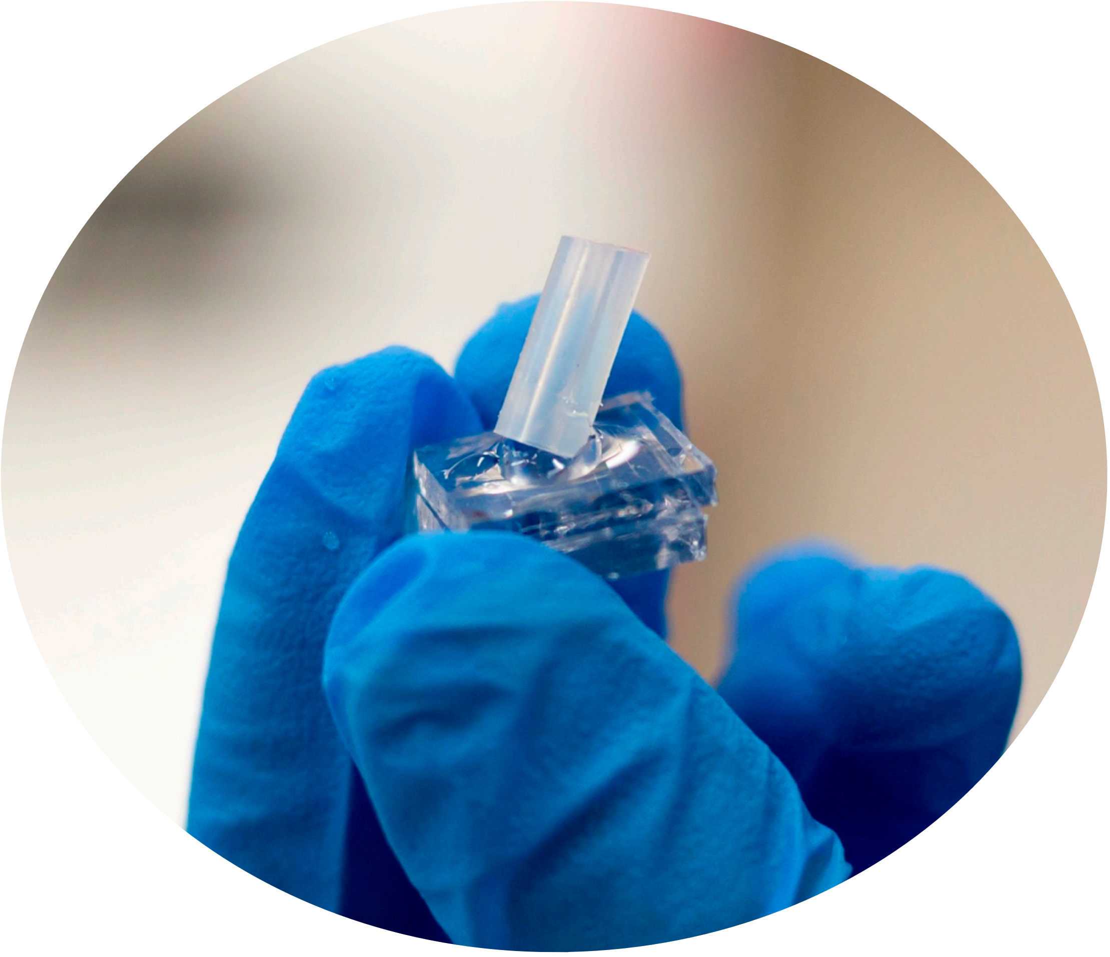

Figure S2. Image of a broken dead-end μF device following the burst pressure test.

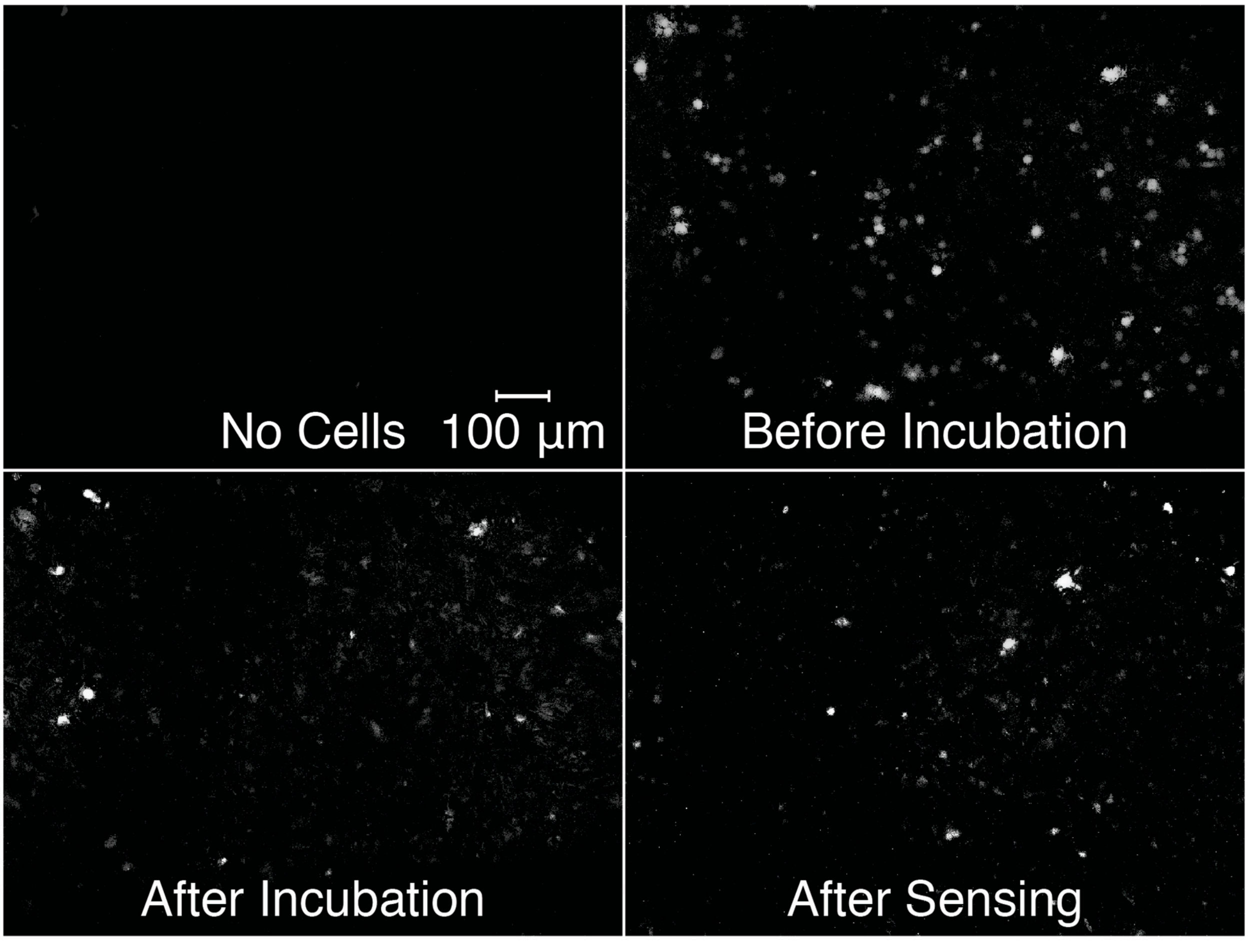

Figure S3. Fluorescence microscope images of the working electrode inside the μF device at different stages of the cell adhesion process compared to the blank electrode (top left). Images were taken of different areas of the electrode. All images were taken at 10x magnification and with identical illumination and acquisition conditions. Bright circular areas represent CFSE labelled cells.

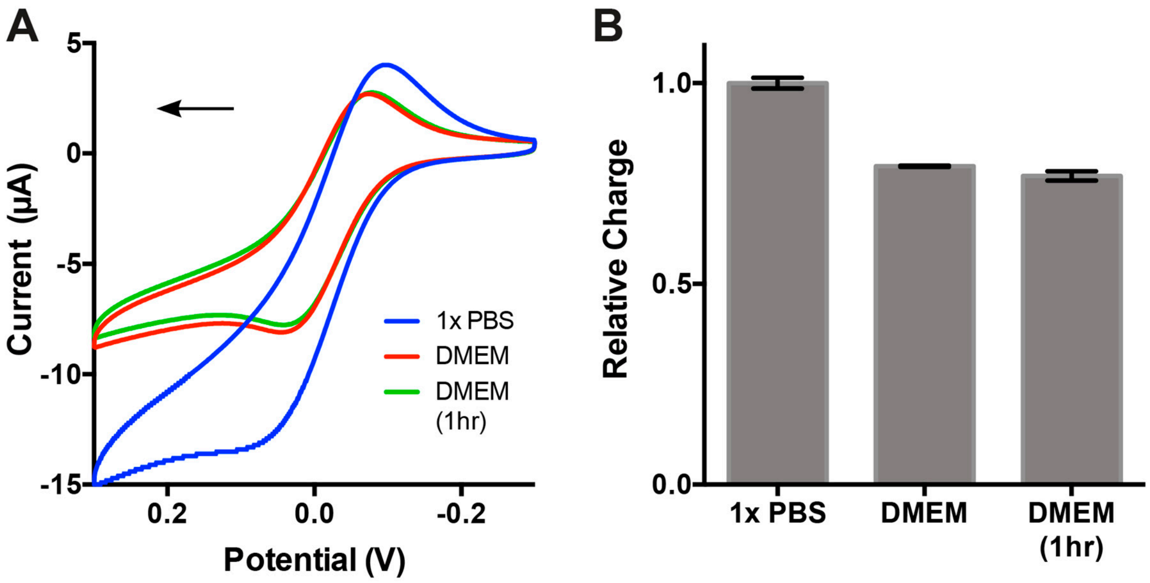

Figure S4. Cyclic voltammogram and relative charge plot comparing electrochemical signals during sensing in 5 mM $K_4[Fe(CN)_6]$ solutions of 1x PBS, DMEM, and DMEM after 1 hour incubation at cell adhesion conditions (37°C and 5% CO_2). (**a**) Overlaid cyclic voltammograms of different control conditions with a positive initial sweep direction as indicated by the arrow. (**b**) Plot comparing relative charge transferred in each condition (normalized to PBS) showing that there is no influence of DMEM on the electrochemical signal before and after 1-hour of incubation. Thus, all electrochemical changes observed during sensing can be attributed to fibroblast cell adhesion.

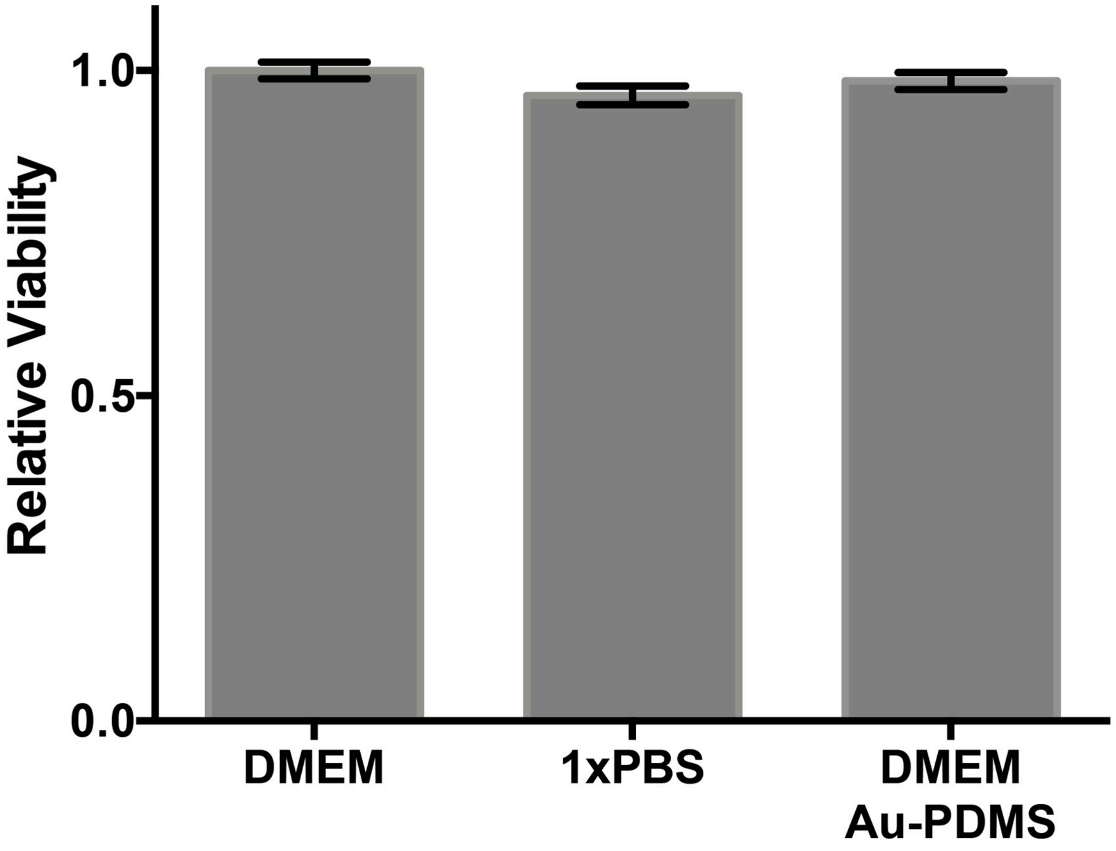

Figure S5. Plot of 3T3 murine fibroblast cells relative viability in 1xPBS, DMEM, and with the Au-PDMS electrode in DMEM. The results show no quantitative difference to cell viability between the different storage conditions.

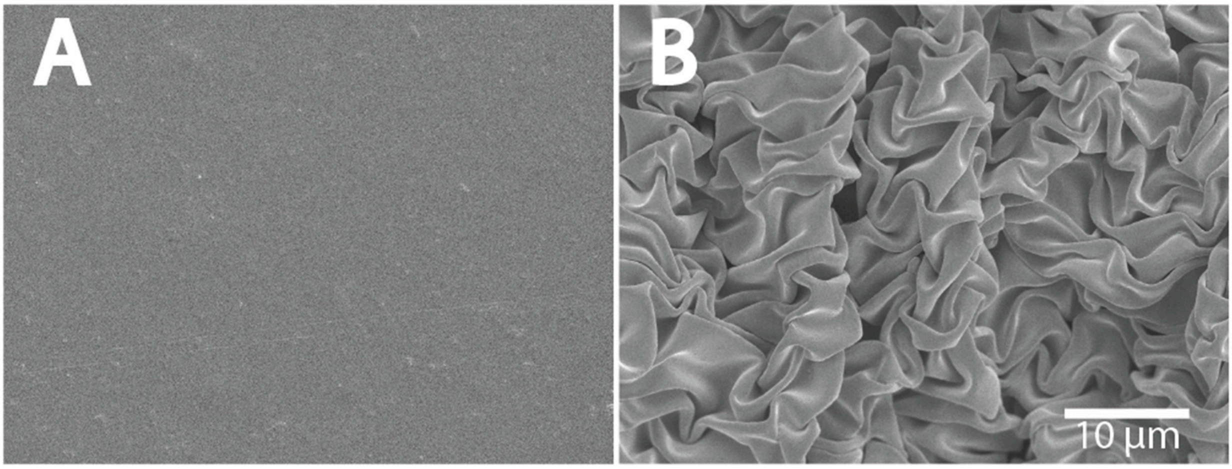

Figure S6. SEM image of gold films before (a) and after (b) shrinking.

Video S1. Video of how the dead-end burst pressure test was performed inside a beaker of water. The dead-end channel was pressurized by N_2 gas and the pressure was monitored until the point of failure.

Video S2. Video demonstrating the laminar flow capability of the µF device in separating the working (red) and reference (green) solutions with food colouring. In this video, the solutions were pumped through the µF device at a flow rate of 0.20 mL/min.

© 2017 by the authors. Submitted for possible open access publication under the terms and conditions of the Creative Commons Attribution (CC-BY) license (http://creativecommons.org/licenses/by/4.0/).
